# Economic Effect of Confiscation of Cattle Viscera Infected with Cystic Echinococcosis, Huancayo Province, Peru

**DOI:** 10.3201/eid2512.181039

**Published:** 2019-12

**Authors:** J. Raúl Lucas, Carmen A. Arias, Stephanie S. Balcázar-Nakamatsu, Alejandro P. Rodríguez, Karen A. Alroy, Cesar M. Gavidia

**Affiliations:** Universidad Nacional Mayor de San Marcos, Lima, Peru (J.R. Lucas, C.A. Arias, S.S. Balcázar-Nakamatsu, C.M. Gavidia);; Servicio Nacional de Sanidad Agraria, Junin, Peru (A.P. Rodríguez);; Johns Hopkins Bloomberg School of Public Health, Baltimore, Maryland, USA (K.A. Alroy)

**Keywords:** bovine, cystic echinococcosis, livers, lungs, slaughterhouse, Peru, Huancayo, Andes, parasites, parasitic infections, zoonoses

## Abstract

We report cystic echinococcosis (CE) prevalence in Huancayo Province, Peru, and the associated economic effect of bovine organ condemnation. CE prevalence during the 16-month study period was 42.8% and caused $14,595 in economic losses. CE threatens food security in the region by reducing farmers’ income and viscera supply in markets.

*Echinococcus granulosus* is a parasitic flatworm found in the small intestines of canids. The metacestode of this parasite (hydatid cyst) can infect various organs of intermediate hosts (mainly livers and lungs), causing cystic echinococcosis (CE) (*1*). The parasite also can infect humans. Intermediate hosts are commonly asymptomatic; however, CE causes economic losses in livestock because of organ condemnation, decreased productivity, and decreased reproductive performance (*2*,*3*). 

Slaughterhouse records have been used as an inexpensive method to record CE data for livestock. These data serve as the foundation for estimating the effects of disease in different endemic regions and can potentially help guide implementation of control programs or serve as an indicator to assess control measures (*4*). We aimed to determine the CE prevalence in cattle slaughtered in a province in the central Andes of Peru and to assess the economic losses and potential food security effects from a multisectoral perspective (e.g., farmers, meat industry, and consumers).

## The Study

We conducted a 2-phased study in 1 of 3 official bovine slaughterhouses (authorized by the Peruvian Ministry of Agriculture) in Huancayo Province (altitude 3,263 m, latitude 12°4′S, longitude 75°13′W), a CE-endemic region of Peru, where prevalence of human CE is >4% (*5*). In this region, cattle are raised primarily for milk production, and the predominant breed is a criollo (i.e., mixed) breed.

In the first phase, we conducted a retrospective review of abattoir meat inspection reports from September 2013–December 2014 to estimate the 16-month offal prevalence and identify the affected organs and sex of the animals. CE-infected organs can be easily distinguished macroscopically, either by palpation and visual inspection (Figure) or, when necessary, by performing incisions in accordance with World Health Organization guidelines (*6*). We evaluated and categorized the records into 4-month periods: September–December 2013, January–April 2014, May–August 2014, and September–December 2014. The cost of noninfected viscera was also recorded from the slaughterhouse register.

**Figure:**
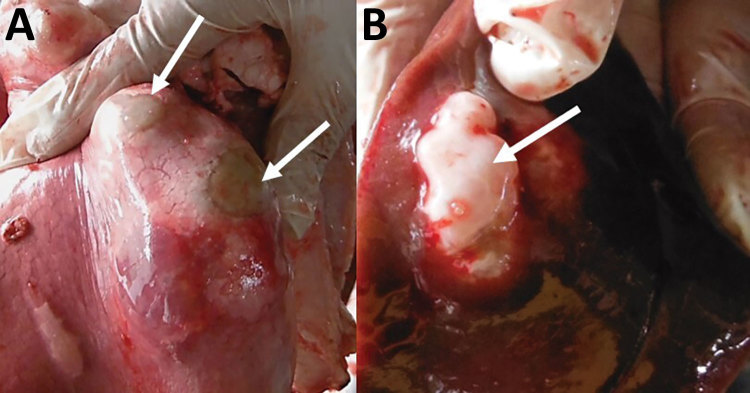
Lung (A) and liver (B) parenchyma infected with hydatid cysts (white arrows) detected during veterinary inspection at slaughterhouse and before the entire organ was confiscated and destroyed by incineration, Huancayo Province, Peru.

In the second phase, initiated in January 2015, we determined the average weight of infected viscera. We then estimated the economic losses by multiplying the number of condemned organs by the average viscera weight and the selling price. In addition, we estimated the total amount of confiscated viscera (expressed as tons of viscera destroyed) for September 2013–December 2014.

We conducted statistical analysis by using Stata 10 (StataCorp, https://www.stata.com). We obtained CE prevalence, 95% CIs, and the prevalence differences between infected organs by χ^2^ test. To estimate the risk for infection, we constructed a multivariate logistic regression model that included as variables the sex of the animal and the month and year of slaughter.

We evaluated data for 7,046 animals during the study period (September 2013–December 2014). The overall 16-month prevalence of CE was 42.8% (95% CI 41.64%–43.96%). We determined specific organ infection by sex and period (Table 1). CE infection of lungs was significantly higher (p<0.001) than in other organs. The sex of the animal and time of year was associated with the presence of CE (Table 2); for example, the odds of CE in >1 infected organ in male animals was 27% lower than that in female animals (p<0.001). In addition, the odds of detecting CE in animals slaughtered during January–April was 3.2 times higher than for May–August 2014 (p<0.001) (Table 2). 

**Table 1:** Prevalence of cystic echinococcosis among cattle and number of infected animals, by 4-month period, sex, and organ type, Huancayo Province, Peru, September 2013–December 2014

Period	% Infected (no. infected animals)							Total prevalence, % (no. infected animals)
	Female				Male			
	Lung	Liver	Heart		Lung	Liver	Heart	
2013 Sep–Dec	48.97 (592)	9.18 (111)	0.17 (2)		41.92 (280)	7.93 (53)	0.15 (1)	49.55 (930)
2014 Jan–Apr	50.46 (383)	14.36 (109)	0.13 (1)		55.19 (351)	12.42 (79)	0.16 (1)	58.64 (818)
2014 May–Aug	32.34 (305)	9.86 (93)	0.32 (3)		24.52 (180)	6.54 (48)	0.14 (1)	31.13 (522)
2014 Sep–Dec	31.98 (433)	21.94 (297)	0.52 (7)		19.52 (145)	15.48 (115)	0.27 (2)	35.72 (749)
2013 Sep–2014 Dec	40.16 (1,713)	14.30 (610)	0.31 (13)		34.38 (956)	17.62 (490)	0.18 (5)	42.85 (3,019)

**Table 2:** Prevalence of cystic echinococcosis among cattle in multivariate logistic regression model, by sex and 4-month period, Huancayo Province, Peru, September 2013–December 2014*

Characteristic	Presence of CE†				Lung CE‡				Hepatic CE†		
	OR (95% CI)		p value		OR (95% CI)		p value		OR (95% CI)		p value
Sex											
F	Referent		NA		Referent		NA		Referent		NA
M	0.73 (0.66–0.81)		0.00		0.75 (0.68–0.83)		0.00		0.73 (0.62–0.84)		0.00
Four-month period§											
Sep–Dec 2013	2.1 (1.85–2.44)		0.00		2.1 (1.82–2.40)		0.00		1.0 (0.80–1.29)		0.89
Jan–Apr 2014	3.2 (2.73–3.69)		0.00		2.8 (2.73–3.20)		0.00		1.7 (1.35–2.15)		0.00
Sep–Dec 2014	1.2 (1.05–1.38)		0.00		0.9 (0.79–1.05)		0.21		2.6 (2.12–3.19)		0.00

*CE, cystic echinococcosis; NA, not applicable; OR, odds ratio. †Pearson χ^2^ goodness-of-fit test value of p<0.05. ‡Pearson χ^2^ goodness-of-fit test value of p = 0.3845. §Reference period is May–August 2014.

Mean weight of affected organs was 2.73 kg (SD + 0.85 kg) for lungs, 4.19 kg (SD + 1.28 kg) for liver, and 1.00 kg (SD + 0.51 kg) for heart. The total weight of destroyed organs during the 16-month period was 11.12 metric tons. The estimated 16-month total economic loss was USD $14,595 (95% CI $12,713–$16,488).

Our results showed that CE infection in slaughtered cattle remains very high in areas like the central Peruvian Andes. Previous reports also described endemic cattle CE in this region with 68% prevalence (17/25 cattle were CE infected) (*5*). With no control program in place in this region of Peru, animal CE has achieved one of the highest infection rates in the world (*4*,*7*–*12*). The large numbers of dogs around the slaughterhouses and traditional human practices are factors that contribute to the high CE infection rates in rural areas (*5*,*7*,*13*).

Pulmonary CE infection was ≈3-fold higher than hepatic CE infection in this study. Multiple studies have indicated that lungs are the most affected organs by CE in ruminants (*5*,*9*,*11*). In contrast, other studies indicate that livers are the most commonly affected organs (*8*,*12*). Although the *E. granulosus* oncospheres first reach hepatic capillaries, lungs have the largest capillary beds in mammals, which might explain the higher prevalence of pulmonary CE infection (*9*). Increased volume and dilation of pulmonary capillaries, associated with the physiologic adaptive response to high altitude in humans (*14*), might also be occurring in cattle in this region.

Similar to our results, other reports have shown that female cattle are more prone to acquire CE than male cattle (*10*,*11*). A biologic explanation for this apparent female susceptibility might exist, but further investigation is required. Husbandry practices in the Andes of Peru might provide another possible explanation because cows commonly remain in production longer than male cattle; consequently, older animals would have a longer exposure time (*4*,*8*,*11*).

May–August is the dry season in the Andes of Peru, and pastures are scarce. Therefore, during this period, slaughtering young cattle instead of older animals is more profitable. This factor would explain the temporal variation observed (Table 2), but concluding that seasonality (e.g., humidity and temperature) plays a role in CE prevalence in the Andes is difficult. However, studies have indicated a higher CE frequency during months of higher humidity in some regions of Iran and Kazakhstan (*10*,*12*).

In South America, the viscera of ≈2 million cattle and ≈3.5 million sheep are condemned in slaughterhouses yearly, which represents a loss of >$6 million USD (*3*). We calculated a 16-month economic loss of $14,595. Previously, the economic loss attributable to ovine and bovine hepatic CE in Peru was reported as $196,681 annually (*13*). Moreover, the loss of livestock to hepatic CE is estimated to be $141,605,195 worldwide (*15*). The economic loss described in our study is from just 1 official abattoir, so when considering farm animal slaughter, unofficial abattoirs, and the remaining slaughterhouses throughout the country, this estimated economic effect might represent just a small portion of the actual effect in Peru. However, we have no information from other slaughterhouses because abattoir information is often unavailable or underestimated. These challenges have been observed in other studies for which similar estimations are performed (*15*).

## Conclusions

The direct economic effects associated with confiscation of infected offal represents only part of the overall losses attributable to CE. Other losses, such as reduction of protein sources and decreased animal productivity, are important components to consider in a global estimation. The central Andes of Peru require interventions aimed at strengthening food security and reducing undernutrition. As demonstrated in this 16-month study, >11 metric tons of viscera were destroyed because of CE infection. Viscera, particularly lungs and livers, are inexpensive sources of protein for human consumption in poor and rural areas of Peru and in other developing countries. Bovine CE infection limits the supply of this protein in local markets and could also result in reduced nutritional quality of carcasses of infected animals (*12*) and in increased prices of suitable noninfected viscera.
